# Construction and external validation of a scoring prediction model for mortality risk within 30 days of community-acquired pneumonia in children admitted to the pediatric intensive care unit: A multicenter retrospective case-control study

**DOI:** 10.1097/MD.0000000000037419

**Published:** 2024-03-08

**Authors:** Xingfeng Cheng, Huizhen Wang, Lingli Sun, Wei Ge, Rui Liu, Hua Qin, Yong Zhang, Changjian Li

**Affiliations:** a Department of Pediatric Intensive Care Unit, Wuhan Children’s Hospital (Wuhan Maternal and Child Healthcare Hospital), Tongji Medical College, Huazhong University of Science and Technology, Wuhan, China; b Department of Neonatal Intensive Care Unit, Wuhan Children’s Hospital (Wuhan Maternal and Child Healthcare Hospital), Tongji Medical College, Huazhong University of Science and Technology, Wuhan, China; c Department of Child Health, Wuhan Children’s Hospital (Wuhan Maternal and Child Healthcare Hospital), Tongji Medical College, Huazhong University of Science and Technology, Wuhan, China; d Department of Pediatrics, Tongcheng People’s Hospital, Xianning, China; e Department of Pediatrics, Macheng People’s Hospital, Huanggang, China; f Department of Pediatrics, Jingmen Second People’s Hospital, Jingmen, China; g Department of Cardiology, Wuhan Children’s Hospital (Wuhan Maternal and Child Healthcare Hospital), Tongji Medical College, Huazhong University of Science and Technology, Wuhan, China.

**Keywords:** binary logistic regression, community-acquired pneumonia, pediatric intensive care unit, risk factors, scoring prediction model

## Abstract

In this study, we constructed and validated a scoring prediction model to identify children admitted to the pediatric intensive care unit (PICU) with community-acquired pneumonia (CAP) at risk for early death. Children with CAP who were admitted to the PICU were included in the training set and divided into death and survival groups according to whether they died within 30 days of admission. For univariate and multifactorial analyses, demographic characteristics, vital signs at admission, and laboratory test results were collected separately from the 2 groups, and independent risk factors were derived to construct a scoring prediction model. The ability of the scoring model to predict CAP-related death was validated by including children with CAP hospitalized at 3 other centers during the same period in the external validation set. Overall, the training and validation sets included 296 and 170 children, respectively. Univariate and multifactorial analyses revealed that procalcitonin (PCT), lactate dehydrogenase (LDH), activated partial thromboplastin time (APTT), and fibrinogen (Fib) were independent risk factors. The constructed scoring prediction model scored 2 points each for PCT ≥ 0.375 ng/mL, LDH ≥ 490 U/L, and APTT ≥ 31.8 s and 1 point for Fib ≤ 1.78 g/L, with a total model score of 0–7 points. When the score was ≥ 5 points, the sensitivity and specificity of mortality diagnosis in children with CAP were 72.7% and 87.5%, respectively. In the external validation set, the sensitivity, specificity, and accuracy of the scoring model for predicting the risk of CAP-related death were 64.0%, 92.4%, and 88.2%, respectively. Constructing a scoring prediction model is worth promoting and can aid pediatricians in simply and rapidly evaluating the risk of death in children with CAP, particularly those with complex conditions.

## 1. Introduction

Community-acquired pneumonia (CAP) is a leading cause of hospitalization and mortality in children aged < 5 years.^[1,2]^ The incidence of CAP among children has substantially declined by approximately 25% as a result of reduction in risk factors for pneumonia and widespread use of pneumonia vaccines over the past decade; nonetheless, CAP remains a major cause of death worldwide.^[3]^ The burden of CAP considerably varies across countries and regions, with 95% of new CAP cases in children aged < 5 years occurring globally each year in developing countries. Of these children, 15% require hospitalization,^[4,5]^ whereas some hospitalized children must be admitted to the pediatric intensive care unit (PICU) for treatment.^[1]^ Despite the availability of guidelines for clinical decision-making, the diagnosis and actual management of childhood pneumonia greatly differ among various regions.^[6–8]^ Consequently, the morbidity and mortality rates of children admitted to the PICU remain high in developing countries owing to differences among regions and other practical measures.^[9,10]^ Accurate identification of children with CAP who are at risk for death, especially in developing countries and poor regions, has enormous implications for the subsequent reduction of child mortality.

Current predictions for childhood pneumonia focus on grading the severity of pneumonia,^[11,12]^ and some previous studies have identified risk factors for CAP-related death in the PICU,^[13,14]^ thereby providing a reference for identifying severe pneumonia by pediatricians. Nevertheless, pneumonia progresses rapidly in children, and the clinical presentation of children with CAP varies widely according to severity.^[15]^ Furthermore, there currently exists a controversy concerning the grading of pneumonia severity for assessment.^[16]^ The commonly accepted prediction score for mortality in children is the Pediatric Index of Mortality 3; however, its calculation is complex and not easily generalizable.^[17]^ When dealing with actual patients, direct identification of children at risk for death using simple, easy-to-use, objective-scoring prediction model markers may be more favorable for subsequent evaluation and treatment. To date, no study has yet reported on a prediction model for the risk of CAP-related mortality in the PICU. Therefore, in the present study, we constructed and validated a scoring prediction model for CAP-related mortality risk in the PICU to help pediatricians in condition assessment.

## 2. Methods

### 2.1. Participants

For the training set, 296 children admitted to the PICU of Wuhan Children’s Hospital from January 2019 to July 2022 who met the inclusion criteria were included in the training set. Among these children, 263 children with CAP (163 males and 100 females; median age: 10.0 [3.0, 26.0] months) survived within 30 days of admission, whereas 33 children with CAP (24 males and 9 females; median age: 13.0 [4.5, 50.5] months) died within 30 days of admission.

For the external validation set, 170 eligible children with CAP who were hospitalized at Tongcheng People’s Hospital, Macheng People’s Hospital, and Jingmen Second People’s Hospital during the same period were included. Among them, 145 children with CAP (86 males and 59 females; median age: 60.0 [21.0, 98.0] months) survived within 30 days of admission, whereas 25 children with CAP (17 males and 8 females; median age: 45.0 [7.5, 101.5] months) died within 30 days of admission.

### 2.2. Diagnostic criteria for CAP

CAP was defined as a pneumonia in a previously healthy child who acquired the infection outside the hospital. This included evidence of acute infection, such as fever, hypothermia, leukocytosis, or leukopenia; evidence of acute respiratory disease, such as new cough or sputum, chest pain, dyspnea, shortness of breath, abnormal pulmonary examination findings, or respiratory failure; and evidence consistent with pneumonia, as assessed using chest radiography or computed tomography prior to admission or within 72 hours of admission. Radiographic evidence of pneumonia included the presence of solid lesions (dense or fluffy opacities with or without signs of bronchial inflation), other infiltrates (linear and patchy alveolar or interstitial densities), and pleural effusion.^[18]^

The inclusion criteria were as follows: children aged between 1 month and 18 years who met the diagnostic criteria for CAP and completed chest radiography or lung computed tomography within 72 hours before and after admission. The exclusion criteria were as follows: children who developed fever or respiratory illness within 14 days prior to admission, had a final diagnosis of immunodeficiency or tuberculosis, lacked data, or abandoned or interrupted the treatment.

### 2.3. Data collection

Data of the study participants were obtained from an inpatient electronic medical record system (Kaihua, Beijing, China). Medical records must provide detailed basic and demographic data of all children, including the hospitalization date, discharge date, discharge outcome, hospitalization number, sex, age, height, weight, and body mass index. Additionally, vital signs at admission, such as the respiratory rate, heart rate, systolic blood pressure, diastolic blood pressure, and oxygen saturation (SpO_2_), were recorded. Laboratory tests were completed on the day of admission and included random blood glucose, blood gas analysis, routine blood examination, biochemical parameters (liver function, kidney function, cardiac enzymes, and electrolytes), coagulation, and infection parameters (high-sensitivity C-reactive protein [hsCRP] and procalcitonin [PCT]). Information obtained from the medical records was recorded by a dedicated professional and carefully proofread by another professional.

This study was approved by the Ethics Committee of Wuhan Children’s Hospital (approval no.: 2022R077). The requirement for the acquisition of informed consent from the participants was waived owing to the retrospective nature of this study.

### 2.4. Treatment protocol

Immediately after the initial diagnosis of CAP was established, the standard treatment for pneumonia (i.e., anti-infective treatment plus supportive therapy) was administered.^[18]^

### 2.5. Laboratory tests

All blood test results of the children were completed on the same day of admission, and the collected blood specimens were sent to the laboratory department for on-machine testing within 2 hours. The test results were checked by a dedicated person in the laboratory department and uploaded to the electronic medical record system. Blood and biochemical tests were regularly calibrated and tested for precision. The coefficient of variation was at the standard limit, and the instruments were controlled daily using high- and low-concentration quality control products.

### 2.6. Statistical analysis

Statistical analysis was performed using SPSS version 23.0 (IBM Corp., New York, NY), with statistical significance being set at *P* < .05. In the training set, the children were first divided into 2 groups (namely, the death and survival groups) according to the presence or absence of death within 30 days of admission. The normal distribution of continuous variables in the 2 groups was assessed using the Shapiro–Wilk test. Comparison of normally distributed variables, expressed as *x* ± s, was conducted between the 2 groups using a *t* test. Differences in non-normally distributed variables, described as median (25th, 75th), were determined between the groups using the Mann–Whitney *U* test. Dichotomous variables were compared between the groups using the chi-squared test and expressed as four-cell tables and composition ratios. Variables showing a significant difference (*P* < .05) between the groups in the univariate analysis were included in the multivariate binary logistic regression analysis. The correlation and strength between the predictor variables in the regression model were measured using the variance inflation factor, in which variance inflation factor < 10 indicated “free of covariance.” Prior to binary logistic regression, the maximum Youden index for continuous variables was calculated using receiver operating characteristic (ROC) curves to determine the cutoff values and subsequently converted to dichotomous variables based on these cutoff values. Variables that were statistically different in the univariate analysis were included in the binary logistic regression equation, and independent risk factors were derived using the backward elimination method. The model was evaluated using the ROC curve, calibration plot (R version 4.3.2, calibrate, MASS package), and Hosmer–Lemeshow test. In order to increase the usefulness of risk stratification in clinical practice, we approximated it using a simple scoring model, in which integer score points were assigned to each variable by rounding the odds ratios (ORs). The total assignment score of each child was calculated. ROC curves were employed to calculate the cutoff score for the entire scoring model and to determine the sensitivity and specificity for predicting the risk of death in children. Finally, the sensitivity, specificity, and accuracy of the scoring model in diagnosing CAP-related death were examined using external validation.

## 3. Results

### 3.1. Demographic characteristics and vital signs on admission in the training set

In the training set, the survival and death groups of children with CAP exhibited no significant differences with respect to sex, age, height, weight, body mass index, systolic blood pressure, diastolic blood pressure, respiratory rate, heart rate, and SpO_2_ (*P* > .05; Table 1).

**Table 1:** Demographic characteristics and admission vital signs in the training set.

Variable(s)	Survival	Death	*Z*/χ^2^	*P* value
Patients (n)	263	33		
Age (mo)	10.0 (3.0–26.0)	13.0 (4.5–50.5)	−1.439	.150
Gender (F/M)	163/100	24/9	1.457	.227
Height (cm)	70.0 (58.0–90.0)	75.0 (64.5–101.5)	−1.493	.135
Weight (kg)	9.0 (5.3–12.0)	10.0 (6.5–14.5)	−1.486	.137
BMI (kg/m^2^)	15.6 (14.1–17.1)	15.3 (13.5–17.9)	−0.279	.780
RR (bpm)	40 (29–48)	36 (27–40)	−1.888	.059
HR (bpm)	138 (120–160)	132 (117–155)	−0.837	.403
SBP (mm Hg)	94.0 (87.0–102.0)	91.0 (81.5–102.0)	−1.347	.178
DBP (mm Hg)	53.0 (48.0–63.0)	52.0 (46.0–57.5)	−1.416	.157
SPO2 (%)	0.92 (0.88–0.96)	0.90 (0.84–0.98)	−1.027	.304

BMI = body mass index, bpm = beats per minute, DBP = diastolic blood pressure, F/M = female/male, HR = heart rate, RR = respiration rate, SBP = systolic blood pressure, SPO2 = oxygen saturation.

### 3.2. Laboratory tests at admission for children with CAP in the training set

#### 3.2.1. Random blood glucose and blood gas analysis.

Blood glucose and blood gas analysis indicators included the following 7 main variables: blood glucose, pH, oxygen pressure (PO_2_), carbon dioxide pressure (PCO_2_), HCO_3_(−), actual bicarbonate (ABE), and lactic acid. HCO_3_(-) and ABE were statistically different between the death and survival groups with CAP (*P* < .05); in particular, both HCO_3_(−) (20.8 vs 22.9 mmol/L, *P* = .024) and ABE (−3.4 vs −1.6, *P* = .014) were lower in the death group than in the survival group (Table 2).

**Table 2:** Laboratory tests on the day of admission of the children with CAP in the training set.

Variable(s)	Survival	Death	*Z*/χ^2^/*t*	*P* value
Patients (n)	263	33		
Random BG and blood gas analysis				
BG (mmol/L)	5.6 (5.0–6.6)	6.1 (5.2–7.3)	−1.150	.250
pH	7.39 (7.34–7.43)	7.38 (7.315–7.41)	−1.432	.152
PO2 (mm Hg)	69.0 (54.0–102.0)	65.0 (50.5–92.5)	−0.866	.386
PCO2 (mm Hg)	39.0 (33.0–47.0)	39.0 (30.7–45.7)	−0.348	.727
HCO_3_(−) (mmol/L)	22.9 (20.0–26.0)	20.8 (16.4–24.8)	−2.251	.024
ABE (mmol/L)	−1.6 (−4.2 to 1.6)	−3.4 (−8.0 to 0.5)	−2.454	.014
Lac (mmol/L)	1.5 (0.9–2.4)	2.1 (1.3–3.3)	−1.950	.051
Blood routine examination and infection indicators				
WBC (10^9/L)	8.64 (5.79–12.63)	6.57 (3.70–14.49)	−0.885	.376
NE (10^9/L)	4.22 (2.20–7.90)	4.71 (1.82–8.47)	−0.206	.837
LYM (10^9/L)	2.65 (1.40–4.21)	1.65 (0.85–4.06)	−2.008	.045
RBC (10^12/L)	3.96 ± 0.77	3.78 ± 0.61	1.266	.207
Hb (g/L)	110.0 (96.0–121.0)	98.0 (87.0–113.5)	−2.347	.019
PLT (10^9/L)	323.0 (229.0–421.0)	201.0 (76.5–358.5)	−2.804	.005
hsCRP (mg/L)	7.86 (1.51–30.00)	24.20 (9.47–47.65)	−2.733	.006
PCT (ng/mL)	0.25 (0.10–1.34)	1.26 (0.50–8.89)	−4.269	<.001
Biochemical indicators and coagulation function				
ALT (U/L)	21 (15–37)	33 (16–96)	−1.905	.057
AST (U/L)	41.0 (30.0–61.0)	97.0 (50.0–217.5)	−4.620	<.001
TP (g/L)	59.9 ± 8.1	56.8 ± 7.9	2.091	.037
ALB (g/L)	40.7 (35.9–44.2)	35.8 (27.4–40.3)	−4.023	<.001
TB (μmol/L)	6.7 (4.8–11.7)	10.9 (6.8–17.1)	−2.625	.009
DB (μmol/L)	3.1 (2.1–4.9)	4.1 (2.4–7.1)	−1.762	.078
Scr (μmol/L)	22.6 (19.0–29.3)	29.8 (23.1–41.8)	−3.563	<.001
BUN (mmol/L)	3.2 (2.3–4.3)	4.5 (2.8–7.9)	−2.698	.007
Na (mmol/L)	138.4 (136.0–140.0)	137.3 (133.6–140.1)	−1.377	.169
K (mmol/L)	4.58 (4.06–5.08)	4.20 (4.00–4.84)	−1.451	.147
CL (mmol/L)	101.0 (98.4–103.7)	101.5 (98.7–105.6)	−0.911	.363
LDH (U/L)	353 (280–523)	692 (461–1166)	−4.761	<.001
CK-MB (U/L)	34.0 (26.0–59.0)	40.0 (20.5–65.5)	−0.079	.937
PT (s)	11.7 (11.0–13.0)	12.7 (11.4–14.7)	−2.659	.008
APTT (s)	32.1 (27.2–38.4)	39.0 (32.8–52.7)	−4.426	<.001
Fib (g/L)	2.6 (1.9–3.7)	2.2 (1.5–2.9)	−2.636	.008

ABE = actual bicarbonate, ALB = albumin, ALT = alanine aminotransferase, APTT = activated partial thromboplastin time, AST = aspartate aminotransferase, BG = blood glucose, BUN = blood urea nitrogen, CAP = community-acquired pneumonia, CK-MB = creatine kinase-MB, CL = chloride ions, DB = direct bilirubin, Fib = fibrinogen, Hb = hemoglobin, hsCRP = high-sensitivity C-reactive protein, K = potassium ions, Lac = lactic acid, LDH = lactate dehydrogenase, LYM = lymphocytes, Na = sodium ions, NE = neutrophils, PCO2 = carbon dioxide pressure, PCT = procalcitonin, PLT = platelets, PO2 = oxygen pressure, PT = prothrombin time, RBC = red blood cells, Scr = serum creatinine, TB = total bilirubin, TP = total protein, WBC = white blood cells.

#### 3.2.2. Routine blood examination and infection indicators.

Routine blood and infection indicators included the following 8 main variables: white blood cells, neutrophils, lymphocytes (LYM), red blood cells, hemoglobin (Hb), platelets (PLT), hsCRP, and PCT. The 2 groups statistically differed in terms of LYM, Hb, PLT, hsCRP, and PCT (*P* < .05). The death group had lower LYM (1.65 × 10^9^/L vs 2.65 × 10^9^/L, *P* = .045), Hb (98.0 vs 110.0 g/L, *P* = .019), and PLT (201.0 × 10^9^/L vs 323.0 × 10^9^/L, *P* = .005) but higher hsCRP (24.2 vs 7.86 mg/L, *P* = .006) and PCT (1.26 vs 0.25 ng/mL, *P* < .001) than the survival group (Table 2).

#### 3.2.3. Biochemical indicators and coagulation function.

Biochemical parameters mainly included the liver function, kidney function, cardiac enzymes, electrolytes, and coagulation indices. Among these, the 10 indicators showing statistical differences were as follows: aspartate aminotransferase (AST), total protein (TP), albumin (ALB), total bilirubin (TB), lactate dehydrogenase (LDH), serum creatinine (SCr), blood urea nitrogen (BUN), prothrombin time (PT), activated partial thromboplastin time (APTT), and fibrinogen (Fib) (*P* < .05). Children in the death group displayed higher AST (97.0 vs 41.0 U/L, *P* < .001), TB (10.9 vs 6.7 μmol/L, *P* = .009), SCr (29.8 vs 22.6 μmol/L, *P* < .001), BUN (4.5 vs 3.2 mmol/L, *P* = .007), LDH (692 vs 353 U/L, *P* < .001), PT (12.7 vs 11.7 s, *P* = .008), and APTT (39.0 vs 32.1 s, *P* < .001) than those in the survival group. Conversely, TP (56.8 vs 59.9 g/L, *P* = .037), ALB (35.8 vs 40.7 g/L, *P* < .001), and Fib (2.2 vs 2.6 g/L, *P* = .008) were lower in the death group than in the survival group (Table 2).

#### 3.2.4. Conversion of continuous variables to dichotomous variables.

Seventeen continuous variables (namely, HCO_3_(−), ABE, LYM, Hb, PLT, hsCRP, PCT, AST, TP, ALB, TB, LDH, SCr, BUN, PT, APTT, and Fib) in the univariate analysis significantly differed between the survival and death groups (*P* < .05). ROC curves were first utilized for clinical use to calculate the area under the curve (AUC), *P* value, and Youden index of each continuous variable for diagnosing CAP-related death. The cutoff values, sensitivity, and specificity corresponding to the maximum Youden index were determined. Subsequently, continuous variables were transformed into dichotomous variables according to the cutoff values (Table 3).

**Table 3:** Conversion of continuous variables to dichotomous variables in the training set.

Cutoff value	AUC (95% CI)	*P* value	Sensitivity	Specificity
HCO_3_(−) ≤ 16.8 mmol/L	0.620 (0.515–0.725)	.024	0.303	0.913
ABE ≤ −5.3	0.631 (0.525–0.737)	.014	0.455	0.825
LYM ≤ 2.19 × 10^9/L	0.607 (0.491–0.723)	.045	0.667	0.593
Hb ≤ 101 g/L	0.625 (0.523–0.728)	.019	0.636	0.650
PLT ≤ 230 × 10^9/L	0.650 (0.537–0.763)	.005	0.606	0.749
hsCRP ≥ 12.4 mg/L	0.646 (0.56–0.732)	.006	0.758	0.578
PCT ≥ 0.375 ng/mL	0.728 (0.645–0.811)	<.001	0.879	0.574
AST ≥ 91 U/L	0.747 (0.651–0.843)	<.001	0.576	0.863
TP ≤ 53.4 g/L	0.629 (0.524–0.733)	.016	0.455	0.810
ALB ≤ 40.3 g/L	0.715 (0.623–0.806)	<.001	0.788	0.532
TB ≥ 7.2 μmol/L	0.640 (0.546–0.735)	.009	0.758	0.540
Scr ≥ 27.6 μmol/L	0.690 (0.597–0.784)	<.001	0.606	0.692
BUN ≥ 3.97 mmol/L	0.644 (0.531–0.757)	.007	0.636	0.669
LDH ≥ 490 U/L	0.754 (0.667–0.841)	<.001	0.758	0.730
PT ≥ 12.7 s	0.642 (0.543–0.741)	.008	0.545	0.711
APTT ≥ 31.8 s	0.736 (0.654–0.819)	<.001	0.879	0.487
Fib ≤ 1.78 g/L	0.641 (0.537–0.745)	.008	0.455	0.814

ABE = actual bicarbonate, ALB = albumin, APTT = activated partial thromboplastin time, AST = aspartate aminotransferase, AUC = the area under the curve, BUN = blood urea nitrogen, CI = confidence interval, Fib = fibrinogen, Hb = hemoglobin, hsCRP = high-sensitivity C-reactive protein, LDH = lactate dehydrogenase, LYM = lymphocytes, PCT = procalcitonin, PLT = platelets, PT = prothrombin time, Scr = serum creatinine, TB = total bilirubin, TP = total protein.

#### 3.2.5. Identification of the independent risk factors and construction of the scoring model.

The transformed dichotomous variables were included in the binary logistic regression equation, with PCT, LDH, APTT, and Fib being identified as independent risk factors. The ORs (95% confidence intervals [CIs]) for diagnosing CAP-related death with PCT ≥ 0.375 ng/mL, LDH ≥ 490 U/L, APTT ≥ 31.8 s, and Fib ≤ 1.78 g/L were 7.378 (2.304–23.628), 4.809 (1.899–12.18), 5.752 (1.837–18.009), and 2.779 (1.101–7.012), respectively.

**Figure 1.**
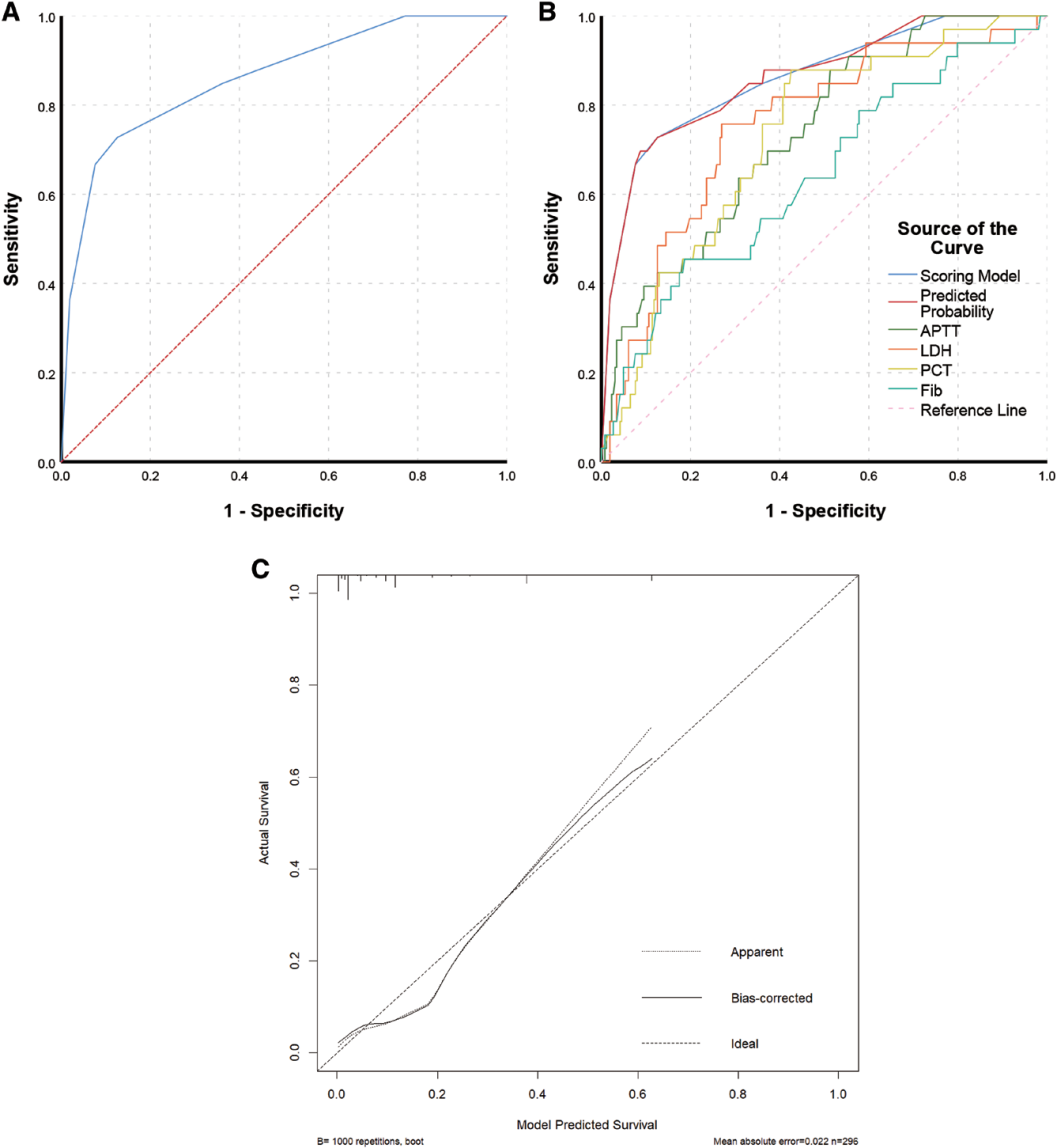
Evaluation of the prediction model. (A) ROC curve of the scoring model. The AUC (95% CI) for predicting CAP-related death was 0.862 (0.789, 0.932) (*P* < .001). (B) ROC curve of the single indicator. The AUC (95% CI) of the predicted probability was 0.862 (0.792, 0.933) (*P* < .001), which was similar to the AUC of the scoring model. The AUC of the scoring model was significantly more significant than the AUC of individual variables, suggesting that the scoring model had a better predictive value than a single indicator. (C) Calibration curve. The diagonal line indicates the ideal reference line where the predicted (x-axis) and observed probability (y-axis) coincide. The closer the bias-corrected line is to the diagonal line, the more accurately the model predicts. AUC = area under the curve, CAP = community-acquired pneumonia, CI = confidence interval, ROC = receiver operating characteristic.

A scoring model was constructed to facilitate its clinical application by approximating the proportion of OR values. We assigned 2 points for PCT ≥ 0.375 ng/mL and 0 point for PCT < 0.375 ng/mL; 2 points for LDH ≥ 490 U/L, and 0 point for LDH < 490 U/L; 2 points for APTT ≥ 31.8 s, and 0 point for APTT < 31.8 s; and 1 point for Fib ≤ 1.78 g/L and 0 point for Fib > 1.78 g/L. The scoring prediction model contained the 4 aforementioned indicators, and the total score was calculated for each child, with each patient receiving a score of 0 to 7 points. Subsequently, for the total score, ROC curves were used to calculate the maximum Youden index corresponding to a cutoff score of 5 points, with an AUC (95% CI) of 0.862 (0.792, 0.932) (*P* ≤ .001). When the model score was ≥ 5 points, the sensitivity and specificity for predicting CAP-related death were 72.7% and 87.5%, respectively (Table 4; Fig. 1A). The AUC (95% CI) of the predicted probability was 0.862 (0.792, 0.933) (*P* < .001), which was similar to the AUC of the scoring model (Fig. 1B).

**Table 4:** Finding independent risk factors and constructing scoring models in the training set.

Variable(s)	*P* value	Odds ratio (95% CI)	Point(s)
PCT ≥ 0.375 ng/mL	.001	7.378 (2.304–23.628)	2
LDH ≥ 490 U/L	.001	4.809 (1.899–12.180)	2
APTT ≥ 31.8 s	.003	5.752 (1.837–18.009)	2
Fib ≤ 1.78 g/L	.030	2.779 (1.101–7.012)	1

APTT = activated partial thromboplastin time, CI = confidence interval, Fib = fibrinogen, LDH = lactate dehydrogenase, PCT = procalcitonin.

#### 3.2.6. Evaluation of the prediction model.

The Hosmer–Lemeshow test showed that the predicted and observed values were not substantially different (*P* > .05), indicating good model suitability. Figure 1C presents the calibration curve. The total score of all patients who died or survived within 30 days in the external validation set was calculated to validate the efficiency of the scoring model. According to the scoring model, patients with a total score of ≥5 or <5 were suspected of death or survival, respectively (Table 5). Based on the scoring model, the outcomes were compared with the patients’ clinical outcomes. The sensitivity, specificity, and accuracy of the scoring model in distinguishing death from survival were 64.0%, 92.4%, and 88.2%, respectively.

**Table 5:** Predictive values of scoring model in the external validation set.

Score (points)	Survival	Death	Total
≥5	11	16	27
<5	134	9	143
Total	145	25	170

## 4. Discussion

PICU admission has a substantial mortality rate for CAP CAP in the PICU has a substantially high mortality rate; therefore, we developed a scoring prediction model to identify children at risk for early CAP mortality. The predictive scoring model comprised 4 independent risk factors: PCT, LDH, APTT, and Fib. We assigned 2 points each for PCT ≥ 0.375 ng/mL, LDH ≥ 490 U/L, and APTT ≥ 31.8 s, and 1 point for Fib ≤ 1.78 g/L, with a total score of 0 to 7 points. When a child’s total score was ≥ 5 points, the sensitivity and specificity for predicting death in children with CAP were 72.7% and 87.5%, respectively. As validated externally in multiple centers, the sensitivity, specificity, and accuracy for predicting CAP-related death were 64.0%, 92.4%, and 88.2%, respectively.

In the prediction model, the first indicator is PCT, which is produced by thyroid C cells and is barely detectable in the blood of healthy individuals but increases during severe bacterial infections, sepsis, and multiorgan dysfunction.^[19]^ In CAP, whether PCT can accurately distinguish bacterial from viral infections controversial.^[20]^ Nonetheless, PCT may reportedly be associated with CAP.^[21–23]^ Our results indicated that the median PCT was significantly lower in the survival group than in the death group (0.25 vs 1.26 ng/mL; *P* < .001). With PCT ≥ 0.375 ng/mL, the sensitivity and specificity for diagnosing CAP-related death were 87.9% and 57.4%, respectively, suggesting that when the PCT index was applied singly, the sensitivity was high, but the specificity was low. Some surviving children were misclassified as dead children. The specificity was improved by constructing a model instead of a single indicator; in the scoring model, PCT ≥ 0.375 ng/mL was scored as 2 points.

The second indicator is LDH, a cytoplasmic enzyme found in all major organ systems^[24]^ that is released into the bloodstream during tissue damage, resulting in an increase in serum LDH levels, which can be used to reflect cellular damage or inflammation.^[25]^ Its predictive value for refractory *Mycoplasma pneumoniae* in children has been previously confirmed,^[26]^ and its role as a risk factor for mortality in adult patients with pneumonia has also been reported.^[27–30]^ Our results confirmed that LDH was significantly higher in the death group than in the survival group (692 vs 353 U/L; *P* < .001). On the regression analysis, the OR (95% CI) for the diagnosis of CAP with LDH ≥ 490 U/L was 4.809 (1.899–12.18), suggesting a 4.809-fold increased risk of death with LDH ≥ 490 U/L compared with LDH < 490 U/L. The sensitivity and specificity of LDH alone for predicting death were 75.8% and 73.0%, respectively, which achieved satisfactory results. This finding suggests that, in clinical practice, pediatricians must pay attention to LDH and be alert to the risk of exacerbation or even death with short-term elevation. LDH ≥ 490 U/L also ended up with a score of 2 points in our scoring model.

The third and fourth indicators in the prediction model were APTT and Fib (coagulation), which are common in CAP and increase with disease severity and poor prognosis.^[31]^ Primary studies have focused on assessing the effects of D2 polymers on the risk of death due to pneumonia.^[32]^ Abnormal coagulation in CAP may be caused by coagulation disorders and inflammation, which are typical host responses to infections in patients with severe sepsis.^[33]^ At the same time, CAP is an important cause of severe sepsis.^[34]^ Our study showed that all coagulation indices in children with CAP who died significantly differed and that PT and APTT were higher in the death group than in the survival group. Additionally, Fib was lower in the death group than in the survival group, indicating that APTT and Fib were independent risk factors for death. The OR (95% CI) for the diagnosis of CAP-related death with APTT ≥ 31.8 s was 5.752 (1.837–18.009), and the sensitivity and specificity for predicting death were 87.9% and 48.7%, respectively. The OR (95% CI) for the diagnosis of CAP-related death with Fib ≤ 1.78 g/L was 2.779 (1.101–7.012), and the sensitivity and specificity for predicting death were 45.5% and 81.4%, respectively. These results suggest that APTT and Fib alone had low specificity or sensitivity for prediction. When these 2 indicators were included in the scoring model, they were scored 2 and 1, respectively. All of the 4 aforementioned clinical indicators (namely, PCT, LDH, APTT, and Fib) correlated with CAP severity and death. A combined prediction with high sensitivity and specificity could be achieved when an OR value was assigned. Ultimately, the sensitivity and specificity of our prediction model for determining CAP-related death in children were 72.7% and 87.5%, respectively, exceeding those of a single predictor. When encountering a complex patient, calculating the relevant score first can be performed as a basis for condition assessment and decision-making.

The present study has some limitations. First, this was a retrospective study involving inpatients with treatment protocols primarily based on diseases, without subgroup interventions. As the PICU admits the most severe CAP in the region, some bias exists, and a multicenter prospective study needs to be refined. Second, the prediction model might require additional sensitivity indicators for refinement to improve its sensitivity. Nevertheless, the significance of our study is that the mortality rate of CAP in the PICU was high, and the early identification of children at risk for death may provide a more substantial basis for physician communication with families, decision-making, and treatment.

## 5. Conclusion

CAP in the PICU has a high mortality rate, and the early identification of children with CAP at risk for death is challenging for PICU physicians. Previous pediatric mortality scores have been complex and controversial. We identified independent risk factors for death due to CAP via a retrospective study and constructed a scoring prediction model that can enable better decision-making among PICU physicians when treating children with CAP.

## Author contributions

**Data curation:** Lingli Sun, Wei Ge, Rui Liu, Hua Qin.

**Methodology:** Yong Zhang.

**Writing – original draft:** Xingfeng Cheng, Huizhen Wang.

**Writing – review & editing:** Changjian Li.

## References

[1] Jain S Williams DJ Arnold SR.; CDC EPIC Study Team. Community-acquired pneumonia requiring hospitalization among U.S. children. N Engl J Med. 2015;372:835–45. 25714161 10.1056/NEJMoa1405870 PMC4697461

[2] GBD 2016 Lower Respiratory Infections Collaborators. Estimates of the global, regional, and national morbidity, mortality, and aetiologies of lower respiratory infections in 195 countries, 1990-2016: a systematic analysis for the Global Burden of Disease Study 2016. Lancet Infect Dis. 2018;18:1191–210. 30243584 10.1016/S1473-3099(18)30310-4 PMC6202443

[3] Rudan I O’Brien KL Nair H.; Child Health Epidemiology Reference Group (CHERG). Epidemiology and etiology of childhood pneumonia in 2010: estimates of incidence, severe morbidity, mortality, underlying risk factors and causative pathogens for 192 countries. J Glob Health. 2013;3:010401. 23826505 10.7189/jogh.03.010401 PMC3700032

[4] Chen K Jia R Li L. The aetiology of community associated pneumonia in children in Nanjing, China and aetiological patterns associated with age and season. BMC Public Health. 2015;15:113. 25879996 10.1186/s12889-015-1422-1 PMC4340102

[5] DeAntonio R Yarzabal JP Cruz JP. Epidemiology of community-acquired pneumonia and implications for vaccination of children living in developing and newly industrialized countries: a systematic literature review. Hum Vaccin Immunother. 2016;12:2422–40. 27269963 10.1080/21645515.2016.1174356 PMC5027706

[6] Florin TA French B Zorc JJ. Variation in emergency department diagnostic testing and disposition outcomes in pneumonia. Pediatrics. 2013;132:237–44. 23878049 10.1542/peds.2013-0179

[7] Brogan TV Hall M Williams DJ. Variability in processes of care and outcomes among children hospitalized with community-acquired pneumonia. Pediatr Infect Dis J. 2012;31:1036–41. 22653486 10.1097/INF.0b013e31825f2b10 PMC3504613

[8] Handy LK Bryan M Gerber JS. Variability in antibiotic prescribing for community-acquired pneumonia. Pediatrics. 2017;139:e20162331. 28270546 10.1542/peds.2016-2331 PMC5369668

[9] Tiewsoh K Lodha R Pandey RM. Factors determining the outcome of children hospitalized with severe pneumonia. BMC Pediatr. 2009;9:15. 19236689 10.1186/1471-2431-9-15 PMC2651138

[10] Lupisan SP Ruutu P Erma Abucejo-Ladesma P.; ARIVAC Consortium. Predictors of death from severe pneumonia among children 2–59 months old hospitalized in Bohol, Philippines: implications for referral criteria at a first-level health facility. Trop Med Int Health. 2007;12:962–71. 17697091 10.1111/j.1365-3156.2007.01872.x

[11] Florin TA Ambroggio L Lorenz D. Development and internal validation of a prediction model to risk stratify children with suspected community-acquired pneumonia. Clin Infect Dis. 2021;73:e2713–21. 33159514 10.1093/cid/ciaa1690 PMC8563216

[12] Williams DJ Zhu Y Grijalva CG. Predicting severe pneumonia outcomes in children. Pediatrics. 2016;138:e20161019. 27688362 10.1542/peds.2016-1019 PMC5051209

[13] Shi T Chen C Huang L. Risk factors for mortality from severe community-acquired pneumonia in hospitalized children transferred to the pediatric intensive care unit. Pediatr Neonatol. 2020;61:577–83. 32651007 10.1016/j.pedneo.2020.06.005

[14] Zhang Q Guo Z Bai Z. A 4 year prospective study to determine risk factors for severe community acquired pneumonia in children in southern China. Pediatr Pulmonol. 2013;48:390–7. 22778084 10.1002/ppul.22608 PMC7168025

[15] Esposito S Cohen R Domingo JD. Antibiotic therapy for pediatric community-acquired pneumonia: do we know when, what and for how long to treat? Pediatr Infect Dis J. 2012;31:e78–85. 22466326 10.1097/INF.0b013e318255dc5b

[16] Dean P Florin TA. Factors associated with pneumonia severity in children: a systematic review. J Pediatric Infect Dis Soc. 2018;7:323–34. 29850828 10.1093/jpids/piy046 PMC6454831

[17] Straney L Clements A Parslow RC.; ANZICS Paediatric Study Group and the Paediatric Intensive Care Audit Network. Paediatric Index of Mortality 3: an updated model for predicting mortality in pediatric intensive care. Pediatr Crit Care Med. 2013;14:673–81. 23863821 10.1097/PCC.0b013e31829760cf

[18] Bradley JS Byington CL Shah SS.; Pediatric Infectious Diseases Society and the Infectious Diseases Society of America. The management of community-acquired pneumonia in infants and children older than 3 months of age: clinical practice guidelines by the Pediatric Infectious Diseases Society and the Infectious Diseases Society of America. Clin Infect Dis. 2011;53:e25–76. 21880587 10.1093/cid/cir531 PMC7107838

[19] Becker KL Nylén ES White JC. Clinical review 167: Procalcitonin and the calcitonin gene family of peptides in inflammation, infection, and sepsis: a journey from calcitonin back to its precursors. J Clin Endocrinol Metab. 2004;89:1512–25. 15070906 10.1210/jc.2002-021444

[20] Kamat IS Ramachandran V Eswaran H. Procalcitonin to distinguish viral from bacterial pneumonia: a systematic review and meta-analysis. Clin Infect Dis. 2020;70:538–42. 31241140 10.1093/cid/ciz545

[21] Don M Valent F Korppi M. Efficacy of serum procalcitonin in evaluating severity of community-acquired pneumonia in childhood. Scand J Infect Dis. 2007;39:129–37. 17366029 10.1080/00365540600951283

[22] Wallihan RG Suárez NM Cohen DM. Molecular distance to health transcriptional score and disease severity in children hospitalized with community-acquired pneumonia. Front Cell Infect Microbiol. 2018;8:382. 30425971 10.3389/fcimb.2018.00382 PMC6218690

[23] Agnello L Bellia C Di Gangi M. Utility of serum procalcitonin and C-reactive protein in severity assessment of community-acquired pneumonia in children. Clin Biochem. 2016;49:47–50. 26386341 10.1016/j.clinbiochem.2015.09.008

[24] Huijgen HJ Sanders GT Koster RW. The clinical value of lactate dehydrogenase in serum: a quantitative review. Eur J Clin Chem Clin Biochem. 1997;35:569–79. 9298346

[25] Drent M Cobben N Henderson RF. Usefulness of lactate dehydrogenase and its isoenzymes as indicators of lung damage or inflammation. Eur Respir J. 1996;9:1736–42. 8866602 10.1183/09031936.96.09081736

[26] Lu A Wang C Zhang X. Lactate dehydrogenase as a biomarker for prediction of refractory mycoplasma pneumoniae pneumonia in children. Respir Care. 2015;60:1469–75. 26060318 10.4187/respcare.03920

[27] Ewig S Bauer T Hasper E. Prognostic analysis and predictive rule for outcome of hospital-treated community-acquired pneumonia. Eur Respir J. 1995;8:392–7. 7789483 10.1183/09031936.95.08030392

[28] Su Y Ju MJ Ma JF. Lactate dehydrogenase as a prognostic marker of renal transplant recipients with severe community-acquired pneumonia: a 10-year retrospective study. Ann Transl Med. 2019;7:660. 31930061 10.21037/atm.2019.10.75 PMC6944597

[29] Sun J Su J Xie Y. Plasma IL-6/IL-10 ratio and IL-8, LDH, and HBDH level predict the severity and the risk of death in AIDS patients with pneumocystis pneumonia. J Immunol Res. 2016;2016:1583951. 27579328 10.1155/2016/1583951 PMC4992515

[30] Reyes S Montull B Martínez R. Risk factors of A/H1N1 etiology in pneumonia and its impact on mortality. Respir Med. 2011;105:1404–11. 21561754 10.1016/j.rmed.2011.04.011

[31] Milbrandt EB Reade MC Lee M.; GenIMS Investigators. Prevalence and significance of coagulation abnormalities in community-acquired pneumonia. Mol Med. 2009;15:438–45. 19753144 10.2119/molmed.2009.00091 PMC2743205

[32] Yang C Zeng HH Huang J. Predictive roles of D-dimer for mortality of patients with community-acquired pneumonia: a systematic review and meta-analysis. J Bras Pneumol. 2021;47:e20210072. 34932717 10.36416/1806-3756/e20210072 PMC8836614

[33] Kinasewitz GT Yan SB Basson B.; PROWESS Sepsis Study Group. Universal changes in biomarkers of coagulation and inflammation occur in patients with severe sepsis, regardless of causative micro-organism [ISRCTN74215569]. Crit Care. 2004;8:R82–90. 15025782 10.1186/cc2459 PMC420030

[34] Kellum JA Kong L Fink MP.; GenIMS Investigators. Understanding the inflammatory cytokine response in pneumonia and sepsis: results of the Genetic and Inflammatory Markers of Sepsis (GenIMS) Study. Arch Intern Med. 2007;167:1655–63. 17698689 10.1001/archinte.167.15.1655 PMC4495652

